# Supplementary material for: Efficient deep-blue electroluminescence from Ce-based metal halide
Source: Nat Commun. 2024 Jul 24;15:6240. doi: 10.1038/s41467-024-50508-5 (PMC11269580; doi:10.1038/s41467-024-50508-5)
Supplement: Supplementary file 1 — Supplementary Information [file 41467_2024_50508_MOESM1_ESM.pdf]

Supplementary Information for

Efficient deep-blue electroluminescence from Ce-based metal halide

Longbo Yang,[1†] Hainan Du,[1†] Jinghui Li,[1] Yiqi Luo,[1] Xia Lin,[1] Jincong Pang,[1] Yuxuan Liu,[1,2] Liang Gao,[1] Siwei He,[3] Jae-Wook Kang,[3] Wenxi Liang,[1,4] Haisheng Song[1], Jiajun Luo,[1*] Jiang Tang[1,4*]

*Corresponding authors. luojiajun@hust.edu; cnjtang@hust.edu.cn.

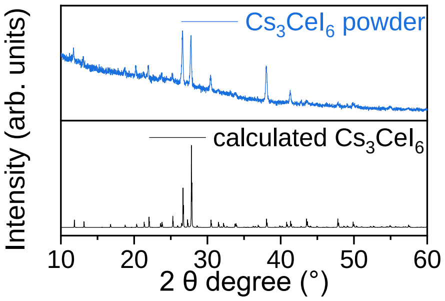

Supplementary Fig. 1 Powder X-ray diffraction spectra of Cs_3CeI_6 crystal

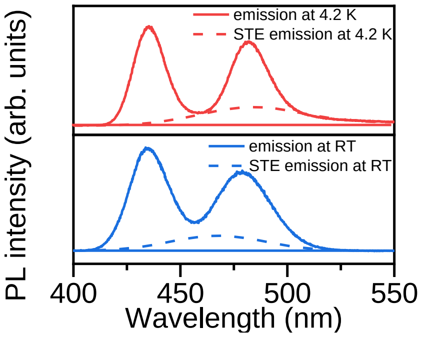

Supplementary Fig. 2 Photoluminescence spectra of Cs_3CeI_6 crystals at room temperature (blue line) and 4.2 K (red line), The dashed lines represent their corresponding STE emission.

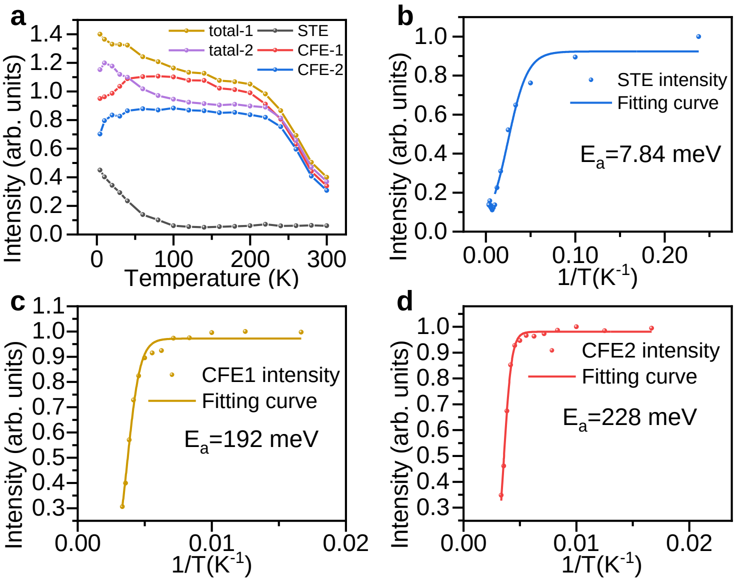

Supplementary Fig. 3 Temperature-dependent PL intensity and activation energy of STE and the two CFE peaks

(a) Temperature-dependent photon-integration PL curves with integrating the STE and the two CFE

peaks in crystal, respectively. CFE1 and CFE2 correspond to the emission at 435 nm and 480 nm, respectively. (b) Fitting diagram of STE activation energy. (c) Fitting diagram of CFE1 activation energy (T>120 K). (d) Fitting diagram of CFE2 activation energy. (T>120 K).

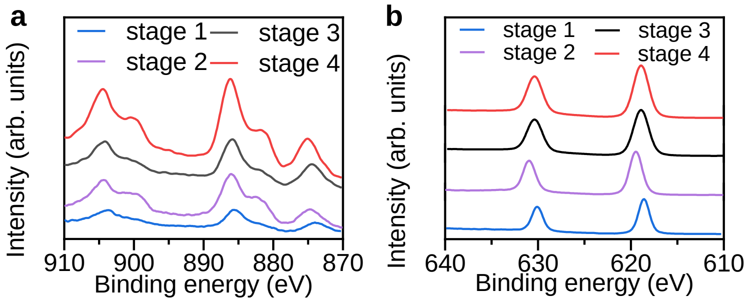

Supplementary Fig. 4 laser–off–on–off–on cycle XPS measurements in-situ

X-ray photoelectron spectroscopy of Ce-3$d_{3/2}$, Ce-3$d_{5/2}$ (a) and I-3$d_{5/2}$ (b) in Cs_3CeI_6 in laser–off–on–off–on cycle. Stage1,2,3,4 stands for ground/excited/ground/excited states. The adjacent stages are tested five minutes apart.

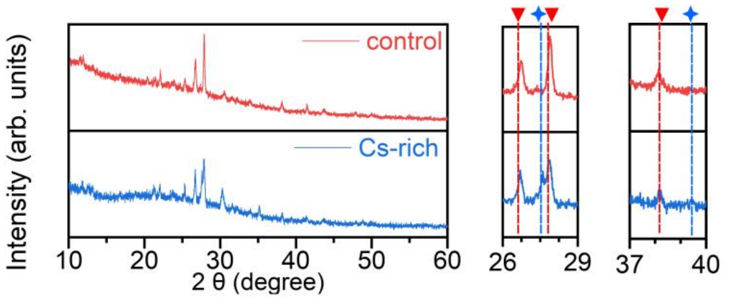

Supplementary Fig. 5 X-ray diffraction spectrum of control Cs_3CeI_6 film (CsI: CeI_3 = 3:1) and the Cs-rich film (CsI: CeI_3 =3.3:1). The blue asterisks represent the XRD peaks of CsI. The red inverted triangle represents the XRD peak of Cs_3CeI_6.

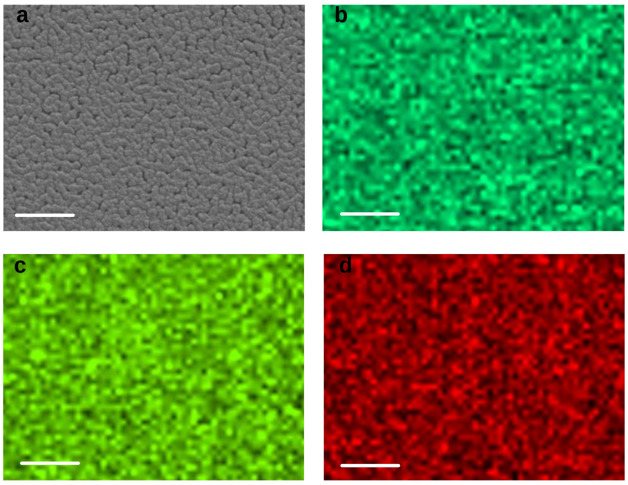

chemical element	Line type	Percentage by weight	Atomic percentage
I	L	58.11	0.595536
Cs	L	31.03	0.303652
Ce	M	10.86	0.100812

Supplementary Fig. 6 The top-view SEM image (a) and EDS mapping (b for Cs, c for I, d for Ce) of the control Cs_3CeI_6 film. (the scale bar in figures is 5μm)

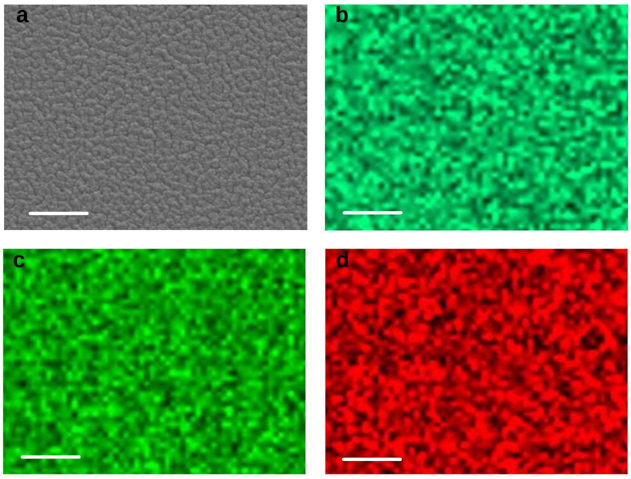

chemical element	Line type	Percentage by weight	Atomic percentage
I	L	56.55	0.579842
Cs	L	33.02	0.323289
Ce	M	10.43	0.096869

Supplementary Fig. 7 The top-view SEM image (a) and EDS mapping (b for Cs, c for I, d for Ce) of the Cs-rich film. (the scale bar in figures is 5μm)

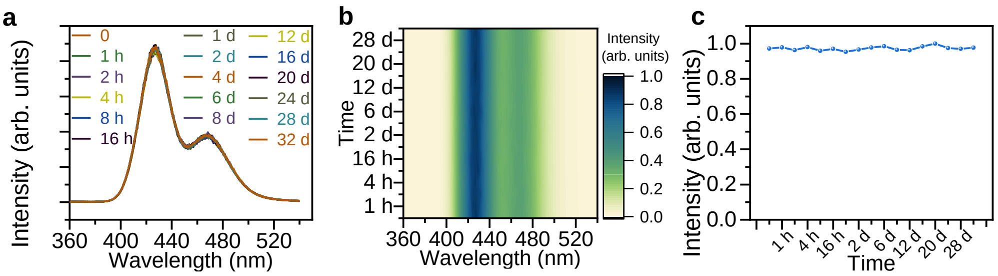

Supplementary Fig. 8 Humidity and thermal stability of Cs_3CeI_6 film

(a, b) The PL spectra of Cs_3CeI_6 film depend on time at 80 °C; (c) The PL intensity of Cs_3CeI_6 film depend on time at 80 °C.

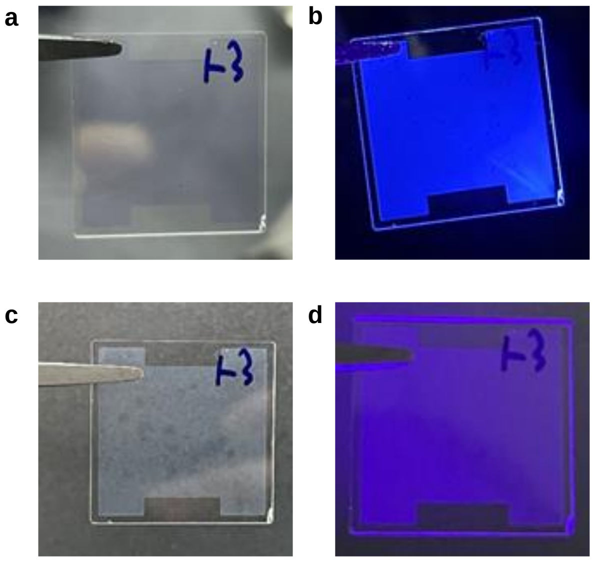

Supplementary Fig. 9 Picture of Cs_3CeI_6 film

(a) in the glovebox; (b) in the glovebox excited with 365 nm light; (c) in the air for several seconds; (d) in the air for several seconds excited with 365 nm light.

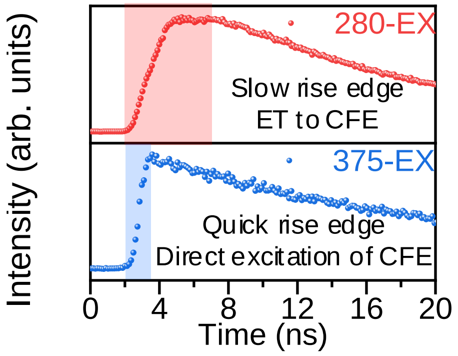

Supplementary Fig. 10 Time-resolved PL decay of Cs_3CeI_6 control film with 280 nm and 375 nm

excitation.

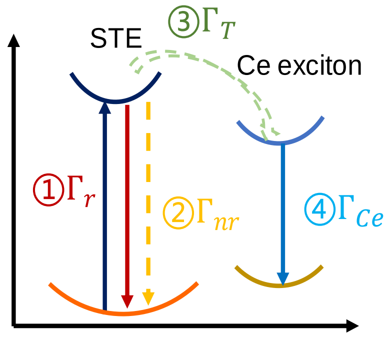

Supplementary Fig. 11 Configuration coordinate diagram of energy transfer process from self-trapped exciton and Ce based Frenkel exciton. Γ_r, Γ_{nr}, Γ_T, represent the radiative, nonradiative, energy transfer decay rate of STE, respectively. Γ_{Ce} represnts the radiative decay rate of Ce exciton.

Supplementary Note 1

Energy transfer model from STE exciton to Ce exciton

The energy transfer process is modeled by two coupled rate equations.

$$\frac{dN_{STE}}{dt} = -(\Gamma_r + \Gamma_{nr} + \Gamma_T)N_{STE} \tag{1}$$

$$\frac{dN_{Ce*}}{dt} = -\Gamma_{Ce}N_{Ce*} \tag{2}$$

N_{STE} is the number of STE located surrounds the Ce exciton. N_{Ce*} is the number of the Ce excited excitons. Γ_r, Γ_{nr}, Γ_T, represent the radiative, nonradiative, energy transfer decay rate of STE, respectively. Γ_{Ce} represnts the radiative decay rate of Ce exciton.

Solving the two rate equations:

$$N_{STE} = N_{STE0}\, e^{-\frac{t}{\tau_E}} \tag{3}$$

$$N_{Ce*} = \frac{N_{STE0}\,\Gamma_T}{\frac{1}{\tau_E} - \frac{1}{\tau_{Ce}}}\left(e^{-\frac{t}{\tau_{Ce}}} - e^{-\frac{t}{\tau_E}}\right) \tag{4}$$

$$\tau_E = \frac{1}{\Gamma_r + \Gamma_{nr} + \Gamma_T} \tag{5}$$

Therefore, the rising edge of 280nm excitation will be different from that of 375nm excitation.

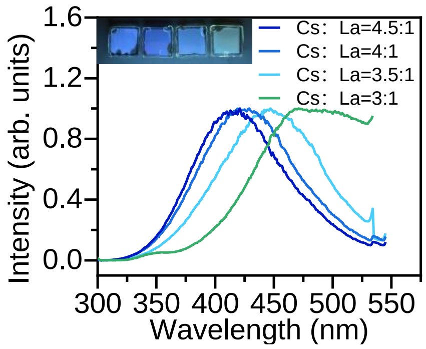

Supplementary Fig. 12 Photoluminescence spectra of the film with different CsI and LaI_3 ratio. (The intensity in the figure has been normalized)

Supplementary Note 2

Calculation of parameters in the energy transfer process

The re-absorption spectral overlap area was calculated by the formula:

$$J(\lambda)=\int_0^{\infty} F_D(\lambda)\varepsilon_A(\lambda)\lambda^4 d\lambda \tag{6}$$

Where $F_D(\lambda)$ and $\varepsilon_A(\lambda)$ represent the STE emission with an integrated intensity normalized to unity and the molar absorption coefficient of CFE respectively, λ represents the wavelength. The overlap area was 4.56×10^{-30} m^3 mol for CsI-rich sample and 1.62×10^{-30} m^3 mol for control sample.

The effective distance of energy transfer R_0 was calculated according to the formula:

$$R_0=2.11\times10^{-2}\times\left[k^2\times J(\lambda)\times\eta^{-4}\times Q_D\right]^{\frac{1}{6}} \tag{7}$$

k^2 represents the spatial orientation factor of the dipole, here we take k^2=2/3, η represents the refractive index of the film, here we take η=1.74, Q_D represents the PLQY of STE. The effective energy transfer distance varies from 3.18 Å to 3.78 Å as the CsI ratio increases.

Energy transfer efficiency was calculated according to the formula:

$$E_T=\frac{1}{1+\left(\frac{r}{R_0}\right)^6} \tag{8}$$

Where r represents the distance between the actual Ce atom and the I atom. We can calculate that the energy transfer efficiency increases from 58.8% to 80.6%.

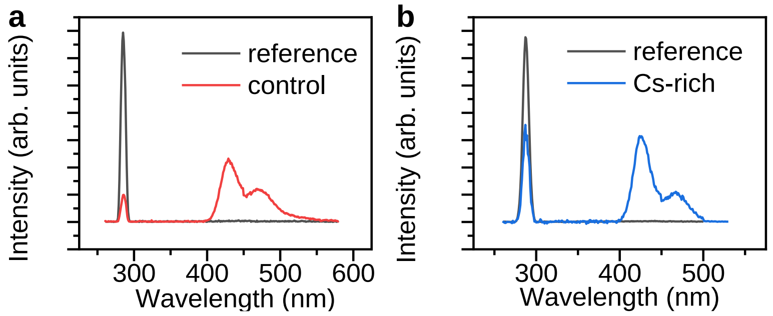

Supplementary Fig. 13 Photoluminescence quantum yield spectra of control (a) and Cs-rich (b) Cs_3CeI_6 film.

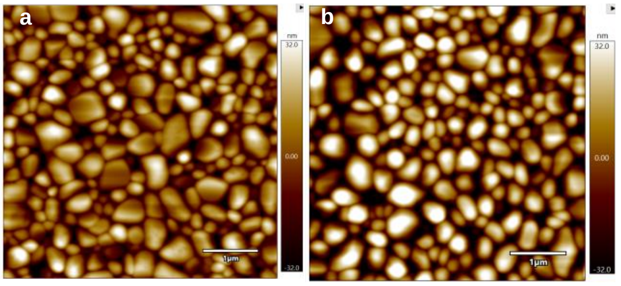

Supplementary Fig. 14 The AFM data for CsI-rich (a) and control films (b).

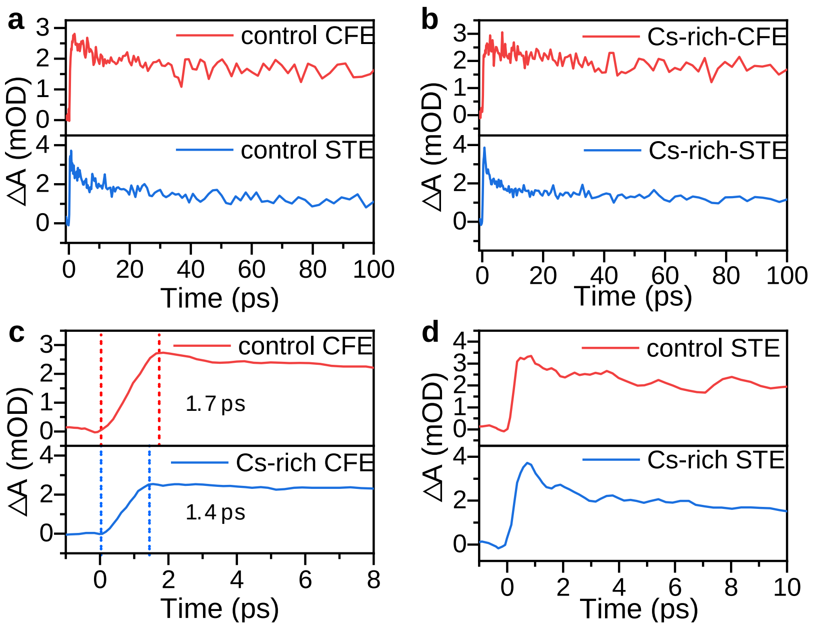

Supplementary Fig. 15 Dynamics of STE and CFE in the control and CsI-rich sample

(a) Versus of transient absorption PIA dynamics signals of CFE excitons and STE excitons in the control sample. (b) Versus of transient absorption PIA dynamics signals of CFE excitons and STE excitons in Cs-rich sample. Their STE signals appeared earlier than the CFE signals. (c) Versus of transient absorption PIA dynamics signals of CFE excitons in control and Cs-rich sample. The 1.7 ps and 1.4 ps in the figure are those rising edge. For a clear rising edge, the data is smoothed. (d) Versus of transient absorption PIA dynamics signals of STE excitons in control and Cs-rich sample. It can be seen that the rich Cs sample has a faster decay within 10 ps compared to the control sample. The pump light is a 250 nm laser with the power of 70 μW when probe light ranges from 320 nm to 580 nm, and 290 μW from 550 nm to 980 nm

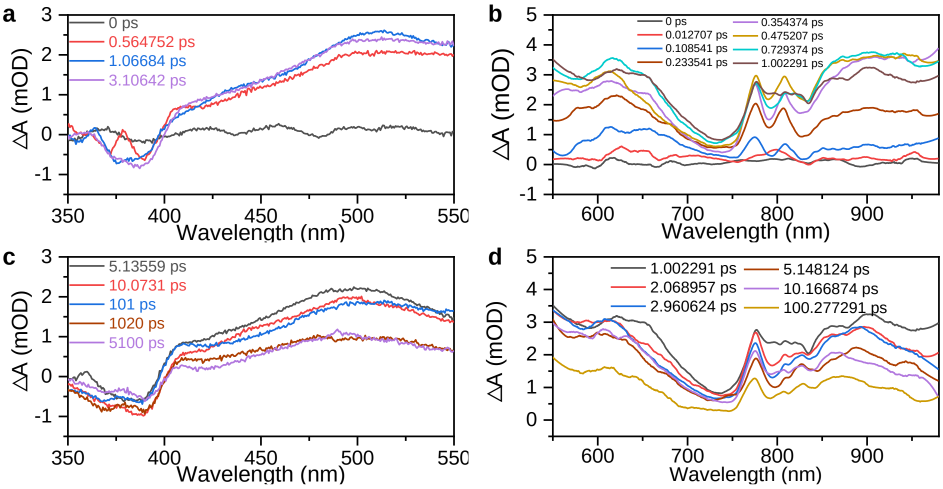

Supplementary Fig. 16 Details of transient absorption data of control sample

The transient absorption spectra of CFE and STE during the rising process at the 350 nm-550 nm band (a) and 550 nm-975 nm band (b); The transient absorption spectra of transient absorption of CFE and STE during the decay process at 350 nm-550 nm band (c) and 550 nm-975 nm band (d);

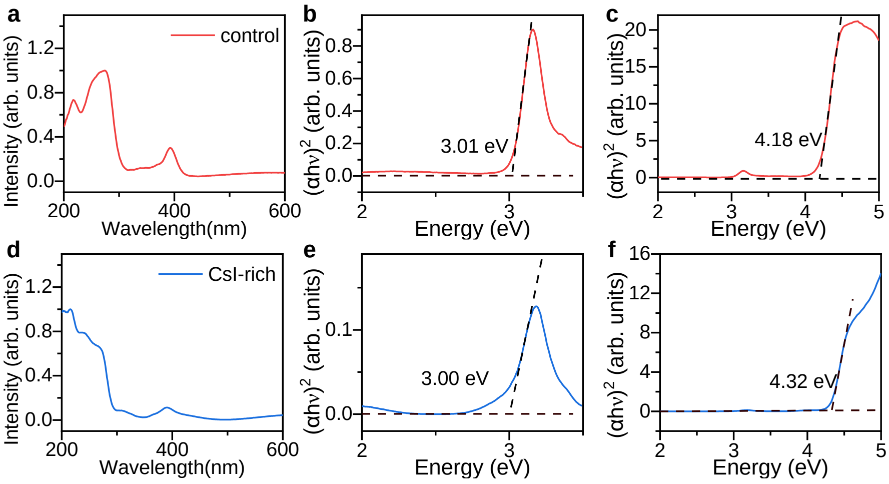

Supplementary Fig. 17 Bandgap fitting of control and CsI-rich thin films

(a-c) Absorption spectra and Optical band-gap fitting of control Cs_3CeI_6 thin films. (d-f) Absorption spectra and Optical band-gap fitting of CsI-rich thin films.

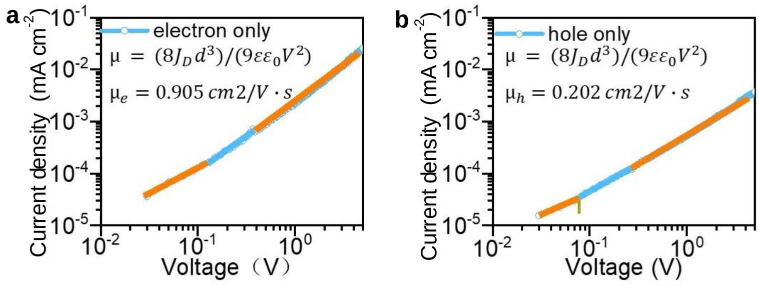

Supplementary Fig. 18 Electron-only and hole-only device

Electron and hole mobility obtained by fitting with space-limited charge current method

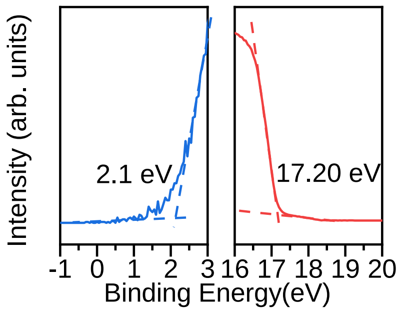

Supplementary Fig. 19 Ultraviolet photoelectron spectroscopy of Cs_3CeI_6 thin films. The energy of the VBM1 is 21.2-17.2+2.1=6.1 eV. The energy of the CBM 6.1-3=3.1 eV. And the energy of the VBM2 is the 3.1+4.3=7.4 eV.

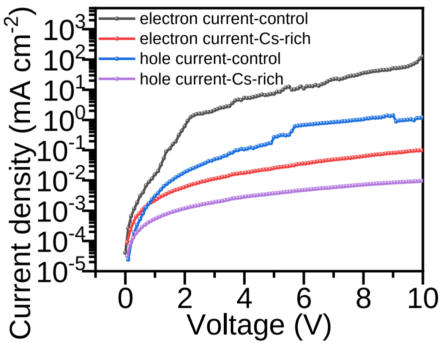

Supplementary Fig. 20 I-V curves of control and Cs-rich single carrier devices

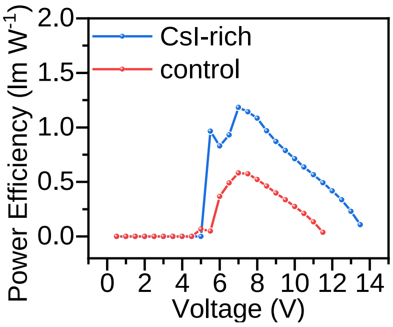

Supplementary Fig. 21 Powder Efficiency-voltage curve of Cs_3CeI_6 control and Cs-rich devices.

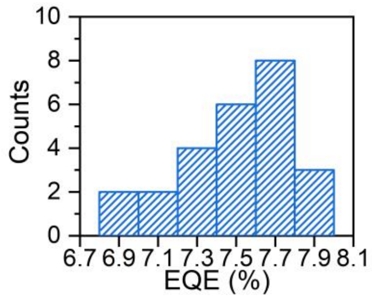

Supplementary Fig. 22 The EQE statistics of Cs-rich Cs_3CeI_6 devices

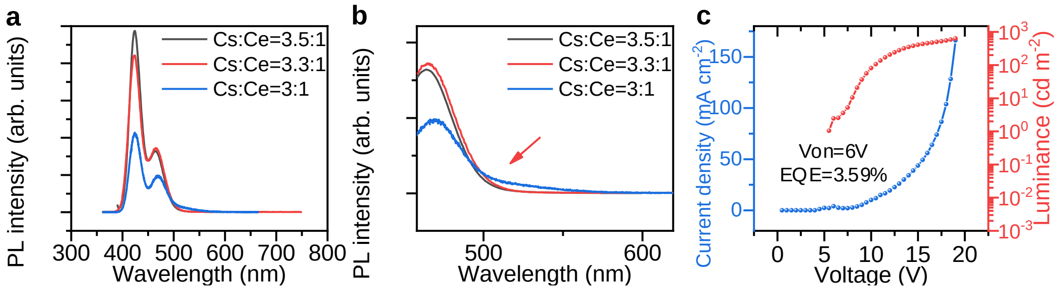

Supplementary Fig. 23 PL spectra and device performance of film with higher Cs/Ce ratio

PL spectra with different Cs:Ce ratio (a) and amplification at STE emission band (b). (c) Current density-voltage-luminance curve with Cs: Ce=3.5:1.

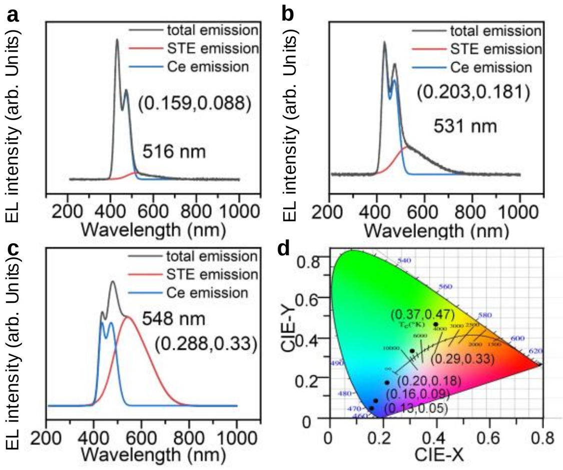

Supplementary Fig. 24 EL spectra of LED with different ratios of CsI and CeI_3

(a) CsI: CeI_3=2.5:1; (b) CsI: CeI_3=2:1; (c) CsI: CeI_3=1.5:1. (d) CIE coordinate diagram of corresponding electroluminescence spectrum. The STE emission spectrum in Cs_3CeI_6 can be redshifted with the increase of Ce ratio, which can be used to build white LEDs

Supplementary Table 1 Summary of representative deep blue metal halide LEDs' performance

**Materials**	**Peak (nm)**	**PLQY %**	**CIE (x, y)**	**EQE %**	**L_{max} (cd m^{-2})**	**Lifetime (min.)**	**Ref.**
$CsPbBr_3$	457	57	(0.141,0.053)	4.62	123.3	90	1
$CsPb(Cl/Br)_3$	456	31.8	(0.151, 0.038)	1.1	43	<1	2
$CsEuBr_3$	448	69	(0.15, 0.04)	6.5	~80	50	3
$Cs_3Cu_2I_5$	445	87	(0.16, 0.07)	1.12	263	6480	4
BI_2PbBr_4	445	80	(0.129, 0.085)	3.08	1315	210	5
$FAPbBr_3$	439	55	(0.161, 0.013)	0.14	9.7	NA	6
$MAPbBr_3$	432	30	(0.231, 0.165)	0.004	~1	NA	7
**Cs_3CeI_6**	**424**	**74.6**	**(0.145 0.057)**	**7.9**	**1073**	**72**	**This work**

Supplementary Table 2 The standard oxidation-reduction potential of Ce^{3+} and halogen

Half reaction	E(V)
$Ce^{4+} + e^- \rightleftharpoons Ce^{3+}$	1.44 eV
$F_2(g) + 2e^- \rightleftharpoons 2F^-$	2.87 eV
$Cl_2(g) + 2e^- \rightleftharpoons 2Cl^-$	1.36 eV
$Br_2(l) + 2e^- \rightleftharpoons 2Br^-$	1.087 eV
$I_2(s) + 2e^- \rightleftharpoons 2I^-$	0.54 eV

Supplementary Table 3 Summary of band gap energy, STE emission energy, and CFE emission energy in different materials

	CsI	Cs-rich	control
Band gap energy	6.20 eV	4.32 eV	4.18 eV
STE emission energy	3.66 eV	2.76 eV	2.57 eV
CFE emission energy	NA	2.87 eV	2.91 eV

Supplementary Table 4 Lifetime of PIA signals at different bands in control and Cs-rich samples

Sample	control		Cs-rich	
PIA signal position	507 nm	880 nm	507 nm	880 nm
Decay time	18 ps	22 ps	24 ps	6 ps

Supplementary References:

1. J. Dong, F. Lu, D. Han, J. Wang, Z. Zang, L. Kong, Y. Zhang, X. Ma, J. Zhou, J. Huiyu, Y. Xuyong, W. Ning, Deep-blue electroluminescence of perovskites with reduced dimensionality achieved by manipulating adsorption-energy differences. *Angew. Chem. Int. Ed.* **61**, e202210322 (2022).
2. H. Chen, L. Zhu, C. Xue, P. Liu, X. Du, K. Wen, H. Zhang, L. Xu, C. Xiang, C. Lin, M. Qin, J. Zhang, T. Jiang, C. Yi, L. Cheng, C. Zhang, P. Yang, M. Niu, W. Xu, J. Lai, Y. Cao, J. Chang, H. Tian, Y. Jin, X. Lu, L. Jiang, N. Wang, W. Huang, J. Wang, Efficient and bright warm-white electroluminescence from lead-free metal halides. *Nat. Commun.* **12**,1421 (2021).
3. J. Luo, L. Yang, Z. Tan, W. Xie, Q. Sun, J. Li, P. Du, Q. Xiao, L. Wang, X. Zhao, G. Niu, L. Gao, S. Jin, J. Tang, Efficient blue light emitting diodes based on europium halide perovskites. *Adv. Mater.* **33**,2101903 (2021).
4. L. T. Wang, Z. F. Shi, Z. Z. Ma, D. W. Yang, F. Zhang, X. Z. Ji, M. Wang, X. Chen, G. R. Na, S. Chen, D. Wu, Y. Zhang, X. J. Li, L. J. Zhang, C. X. Shan, Colloidal synthesis of ternary copper halide nanocrystals for high-efficiency deep-blue light-emitting diodes with a half-lifetime above 100 h. *Nano Lett.* **20**,3568–3576 (2020).
5. S. Yan, W. Tian, H. Chen, K. Tang, T. Lin, G. Zhong, L. Qiu, X. Pan, W. Wang, Deep blue layered lead perovskite light-emitting diode. *Adv. Opt. Mater.* **9**,2001709 (2020).
6. S. Peng, Z. Wen, T. Ye, X. Xiao, K. Wang, J. Xia, J. Sun, T. Zhang, G. Mei, H. Liu, B. Xu, X. Li, R. Chen, G. Xing, K. Wang, Z. Tang, Effective surface ligand-concentration tuning of deep-blue luminescent $FAPbBr_3$ nanoplatelets with enhanced stability and charge transport. *ACS Appl. Mater. Interfaces* **12**, 31863–31874 (2020).
7. S. Kumar, J. Jagielski, S. Yakunin, P. Rice, Y.-C. Chiu, M. Wang, G. Nedelcu, Y. Kim, S. Lin, E. J. G. Santos, M. V. Kovalenko, C.-J. Shih, Efficient blue electroluminescence using quantum-confined two-dimensional perovskites. *ACS Nano* **10**, 9720–9729 (2016).
